# Differentiation of Central Lung Cancer from Atelectasis: Comparison of Diffusion-Weighted MRI with PET/CT

**DOI:** 10.1371/journal.pone.0060279

**Published:** 2013-04-04

**Authors:** Rui-Meng Yang, Long Li, Xin-Hua Wei, Yong-Mei Guo, Yun-Hai Huang, Li-Sha Lai, A-Mei Chen, Guo-Shun Liu, Wei-Feng Xiong, Liang-Ping Luo, Xin-Qing Jiang

**Affiliations:** 1 Department of Radiology, Guangzhou First People’s Hospital, Guangzhou Medical College, Guangzhou, China; 2 Department of Radiology, Guangdong Provincial Corps Hospital, Chinese People’s Armed Police, Guangzhou, China; 3 Department of Radiology, The First Clinic Medical College, Jinan University, Guangzhou, China; Cincinnati Children’s Hospital Medical Center, United States of America

## Abstract

**Objective:**

Prospectively assess the performance of diffusion-weighted magnetic resonance imaging (DW-MRI) for differentiation of central lung cancer from atelectasis.

**Materials and Methods:**

38 consecutive lung cancer patients (26 males, 12 females; age range: 28–71 years; mean age: 49 years) who were referred for thoracic MR imaging examinations were enrolled. MR examinations were performed using a 1.5-T clinical scanner and scanning sequences of T1WI, T2WI, and DWI. Cancers and atelectasis were measured by mapping of the apparent diffusion coefficients (ADCs) obtained with a b-value of 500 s/mm^2^.

**Results:**

PET/CT and DW-MR allowed differentiation of tumor and atelectasis in all 38 cases, but T2WI did not allow differentiation in 9 cases. Comparison of conventional T2WI and DW-MRI indicated a higher contrast noise ratio of the central lung carcinoma than the atelectasis by DW-MRI. ADC maps indicated significantly lower mean ADC in the central lung carcinoma than in the atelectasis (1.83±0.58 *vs.* 2.90±0.26 mm^2^/s, *p*<0.0001). ADC values of small cell lung carcinoma were significantly greater than those from squamous cell carcinoma and adenocarcinoma (*p*<0.0001 for both).

**Conclusions:**

DW-MR imaging provides valuable information not obtained by conventional MR and may be useful for differentiation of central lung carcinoma from atelectasis. Future developments may allow DW-MR imaging to be used as an alternative to PET-CT in imaging of patients with lung cancer.

## Introduction

Central bronchogenic tumors often cause post-obstructive pneumonia resulting in lung volume loss that can induce atelectasis [1]. Atelectasis and tumors both appear as solid dense shadows on standard radiography, so differentiation can be difficult. However, accurate characterization of the tumor is important for clinical staging, and differentiation of the tumor mass from atelectasis is important for CT-guided biopsy, setting of the radiation field for radiotherapy, and evaluation of therapeutic results. Therefore, computed tomography (CT) and magnetic resonance imaging (MRI) are often used together to differentiate lung cancer and atelectasis [2]–[5]. New diagnostic approaches, such as the integration of MRI and PET images through image fusion software, are also becoming more popular [6]. However, this method can be expensive, is not routinely available, and involves exposure to additional radiation.

Rapid improvements in the hardware and software used for MRI have led to new potential approaches for MRI-based pulmonary imaging, such as diffusion-weighted (DW)-MRI. In DW-MRI, image contrast is a function of the rate of water diffusion, as indicated by the apparent diffusion coefficient (ADC), and this allows differentiation of normal and pathological tissues. DW-MRI has well-known clinical utility for the evaluation of intracranial diseases, such as early cerebral ischemia, white matter disorders, epilepsy, depression, dementia, and other brain diseases [7], [8]. Furthermore, developments of echo-planar imaging (EPI), high gradient amplitudes, multi-channel coils, and parallel imaging have reduced image distortion and increased the signal-to-noise ratio (SNR), making whole-body DW-MRI more feasible.

The purpose of our current investigation was to prospectively evaluate the performance of DW-MRI in the differentiation of central lung carcinoma and accompanying atelectasis.

## Materials and Methods

### Patient Characteristics

The ethics committee of Guangzhou First People’s Hospital, Guangzhou Medical College approved this study and all patients provided written informed consent. From March 2009 to June 2012, 38 consecutive patients (26 males, 12 females; age range: 28–71 years; mean age: 49 years) from the radiology departments of three institutions: *(i)* Guangdong Provincial Corps Hospital of Chinese People’s Armed Police; *(ii)* Guangzhou First People’s Hospital of Guangzhou Medical College; and *(iii)* First Clinic Medical College of Jinan University were enrolled. These patients(met the following inclusion criteria: *(i)* pathological diagnosis of lung cancer; *(ii)* indication of existing central lung cancer and atelectasis based on PET-CT; *(iii)* no receipt of chemotherapy, radiotherapy, or other oncological therapy; *(iv)* no contraindication for MRI scans.

### PET-CT Imaging

After fasting for 4–6 h, all patients were given intravenous 2-deoxy-2-(^18^F)fluoro-D-glucose (FDG, 744±39.2 MBq, 20.10±1.05 mCi). An uptake phase of 60 min was used prior to imaging. All patients were encouraged to void before scanning. Images were obtained from the head to the proximal thighs with a combined PET/CT scanner (Discovery ST; General Electric Medical Systems, Milwaukee, WI). The unenhanced CT scan first was performed from the patient’s head to the proximal thighs. Immediately after this, and without changing the patient’s position, the tabletop moved automatically to the PET position. This scan was acquired starting at the mid thighs toward the head, for 6–7 bed positions of 4 min each. CT images were used to generate the transmission maps for attenuation correction of the PET acquisitions. PET data were reconstructed using an ordered-subset expectation maximization iterative algorithm as instructed by the PET device.

### Thoracic MR Imaging

All MRI examinations were performed with a clinical scanner (Signa Infinity with EXCITE 1.5T System, GE Healthcare, Milwaukee, WI, USA) using a four-channel body phased-array coil. MRI was performed within 1–2 weeks after PET-CT while patients were still hospitalized. All patients were in the supine position throughout examinations. The MR scanning sequences were SE T1WI, FRFSE T2WI, FIESTA, and DW-MRI. All sequences used respiratory gating and electrocardiogram gating. T1WI was obtained with the spin echo sequence with the following parameters: repetition time/echo time: 705.08 ms/15.00 ms; number of excitations: 2; direction of frequency encoding: R/L; section thickness: 8 mm; gap: 0.5 mm; field of view: 36–40 cm; matrix: 288×224. T2WI was obtained with the FRFSE sequence with the following parameters: repetition time/echo time, 6000 ms/89.70 ms; number of excitations, 3; direction of frequency encoding: R/L; section thickness, 8 mm; gap, 0.5 mm; field of view, 36∼40 cm; matrix, 288×224. T2WI was obtained with the FIESTA sequence using the following parameters: repetition time/echo time: 3.38 ms/1.49 ms; number of excitations: 3; direction of frequency encoding: R/L; section thickness: 8 mm; gap: 0.5 mm; field of view: 36∼40 cm; matrix: 288×224. DW-MRI was performed with a single-shot (SS) spin-echo (SE) echo-plannar imaging (EPI) sequence with an array spatial sensitivity encoding technique (ASSET) in the axial plane during breath-holding. Images were obtained at a b value of 500 s/mm^2^ for each section in the same sequence using the following parameters: TR/TE: 4000 ms/64.9 ms; number of excitations: 4; section thickness: 7 mm; intersection gap: 0.5 mm; field of view: 40 cm; matrix size: 128×128; diffusion gradient encoding: 3 orthogonal directions.

### Imaging Evaluation and Diagnostic Criteria

All PET/CT and MR images were independently analyzed by two senior radiologists. If these radiologists disagreed, a third senior radiologist was consulted until a consensus was reached. Image analysis involved assessment and comparison of FDG uptake (PET/CT images) and signal density (MR images) of central lung carcinoma and atelectasis.

Distinguishable cases are those in which differentiation of lung tumor and atelectasis was possible, and indistinguishable cases are those in which differentiation was not possible. In the evaluation of DW-MR images, cases in which the tumor had obvious hyper-intensity relative to the atelectasis were considered distinguishable, and cases in which the signal of the atelectasis was continuous with the tumor were indistinguishable.

In each patient, evaluation of conventional MR and DW-MR images was performed with regions of interest (ROIs) from three different locations. ROIs were drawn and placed on the masses, atelectasis, and background, with exclusion of necrotic areas. Signal intensities of the ROIs were measured, and the contrast-to-noise ratio (CNR) of the tumor and atelectasis on conventional T2W and DW-MR images were calculated according to the following equation: CNR = |SI_cancer_-SI_atelectasis_|/SD where SI_cancer_ is the signal intensity of central lung cancer, SI_atelectasis_ is the signal intensity of the atelectasis, and SD is the standard deviation of the background noise.

The ADC maps were automatically reconstructed for all DW-MR images by the GE AW4.2 workstation. ADC values of each ROI were also measured on the ADC maps.

### Statistical Analysis

All numerical data are presented as box plots. The Mann-Whitney test was used for comparisons of CNR values from conventional T2W and DW-MR images, mean ADC values of the cancer and atelectasis, and ADC values of different histological types of lung cancers. All data were analyzed using SPSS 15.0 software (SPSS Inc, Chicago, IL, US), and a *p*-value less than 0.05 was considered statistically significant.

## Results

### Clinical Characteristics of Patients

Table 1 summarizes the clinical characteristics of the 38 enrolled lung cancer patients. A lesion was present in the left upper lobe of 16 patients, in the left inferior lobe of 8 patients, in the right upper lobe of 9 patients, and in the right inferior lobe of 5 patients. Sixteen patients had squamous cell carcinoma, 13 patients had adenocarcinoma, and 9 patients had small cell lung carcinoma. Histological diagnosis was obtained by trans-bronchial aspiration biopsy in 26 cases and by CT-guided biopsy in 12 cases.

**Table 1:** Clinical characteristics of 38 enrolled lung cancer patients.

		Mean ± SD (range) or n (%)
Age (years)		49±9.8 (29–71)
Sex	Male	26 (68%)
	Female	12 (31%)
Histological type	Squamous cell lung carcinoma	16 (42%)
	Adenocarcinoma	13 (34%)
	Small cell lung carcinoma	9 (23%)
Clinical stage	Stage I	10 (26%)
	Stage II	18 (47%)
	Stage III	4 (10%)
	Stage IV	6 (15%)

### PET/CT and MR Imaging of Central Lung Carcinoma and Atelectasis

**Figure 1:**
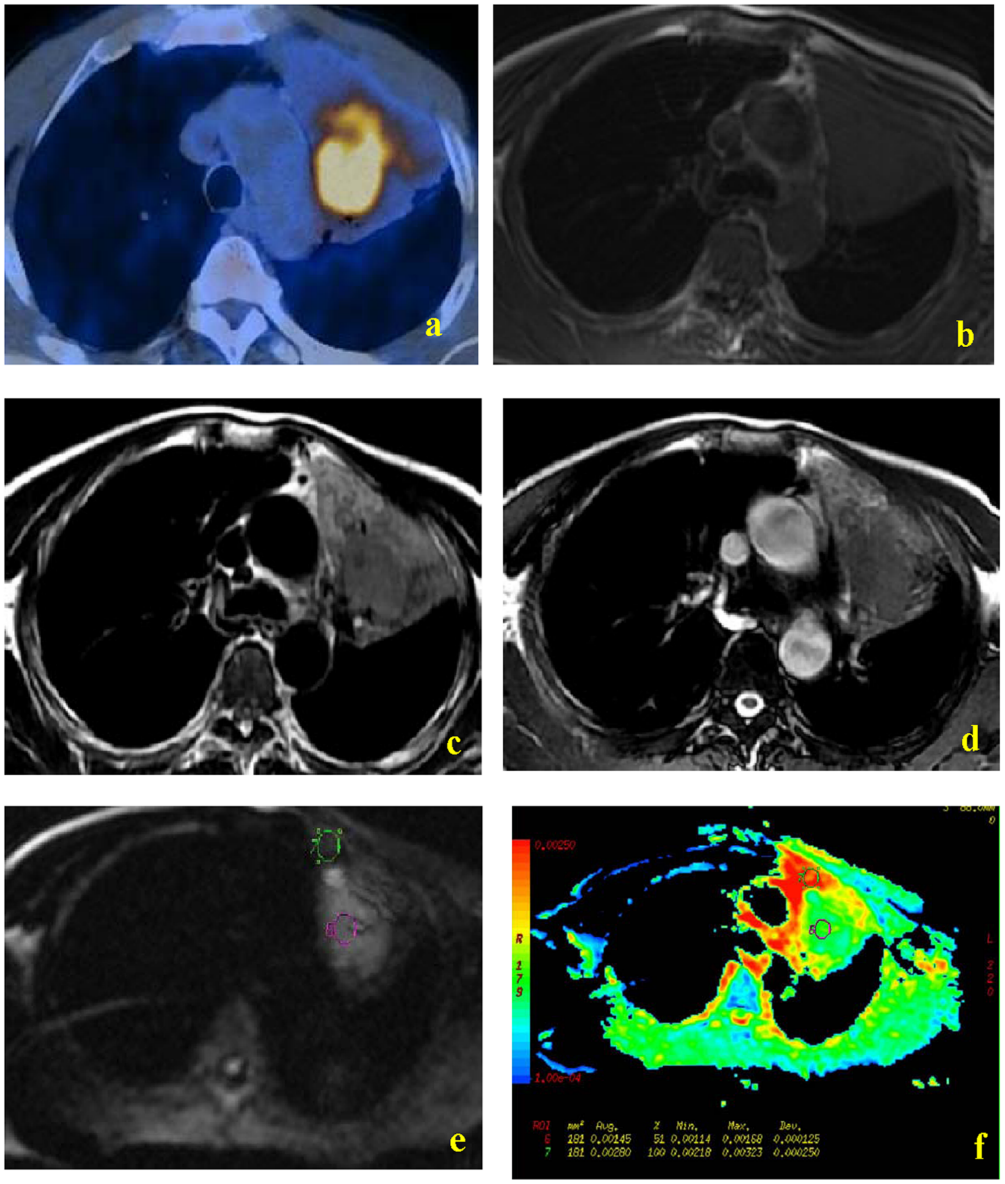
MR and PET/CT images of a 39-year-old woman with lung adenocarcinoma. PET-CT indicated greater uptake of FDG by tumor than atelectasis (a). T1W MR imaging showed a soft-tissue shadow in the left upper lobe, but similar signal intensities of the central lung carcinoma and the distal atelectasis were noted (b). FRFSE T2W images and FIESTA images indicated hypointensity of the tumor mass relative to the atelectasis (c and d, respectively). DW images obtained with a diffusion gradient of 500 s/mm^2^ allowed easy differentiation of tumor and atelectasis (e and f).

**Figure 2:**
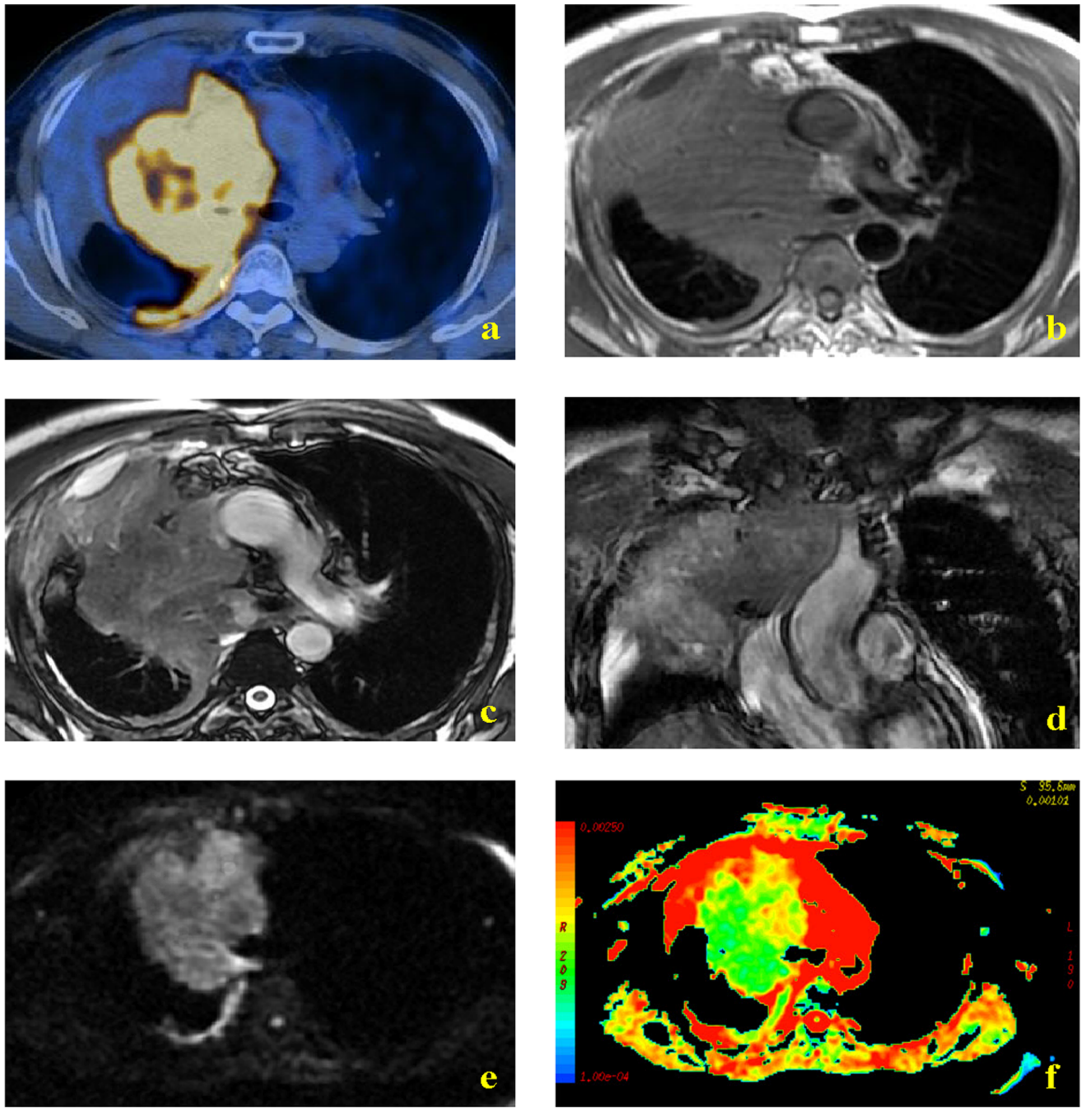
MR and PET/CT images of a 58-year-old man with small cell lung carcinoma. PET-CT indicated greater uptake of FDG by tumor than atelectasis (a). T1W MR imaging showed a soft-tissue shadow in the right upper lobe, but similar signal intensities of the central lung carcinoma and the distal atelectasis were noted (b). FRFSE T2W axial and coronal images indicated hypointensity of the tumor mass relative to the atelectasis (c and d, respectively). DW images obtained with a diffusion gradient of 500 s/mm^2^ allowed easy differentiation of tumor and atelectasis (e and f).

**Figure 3:**
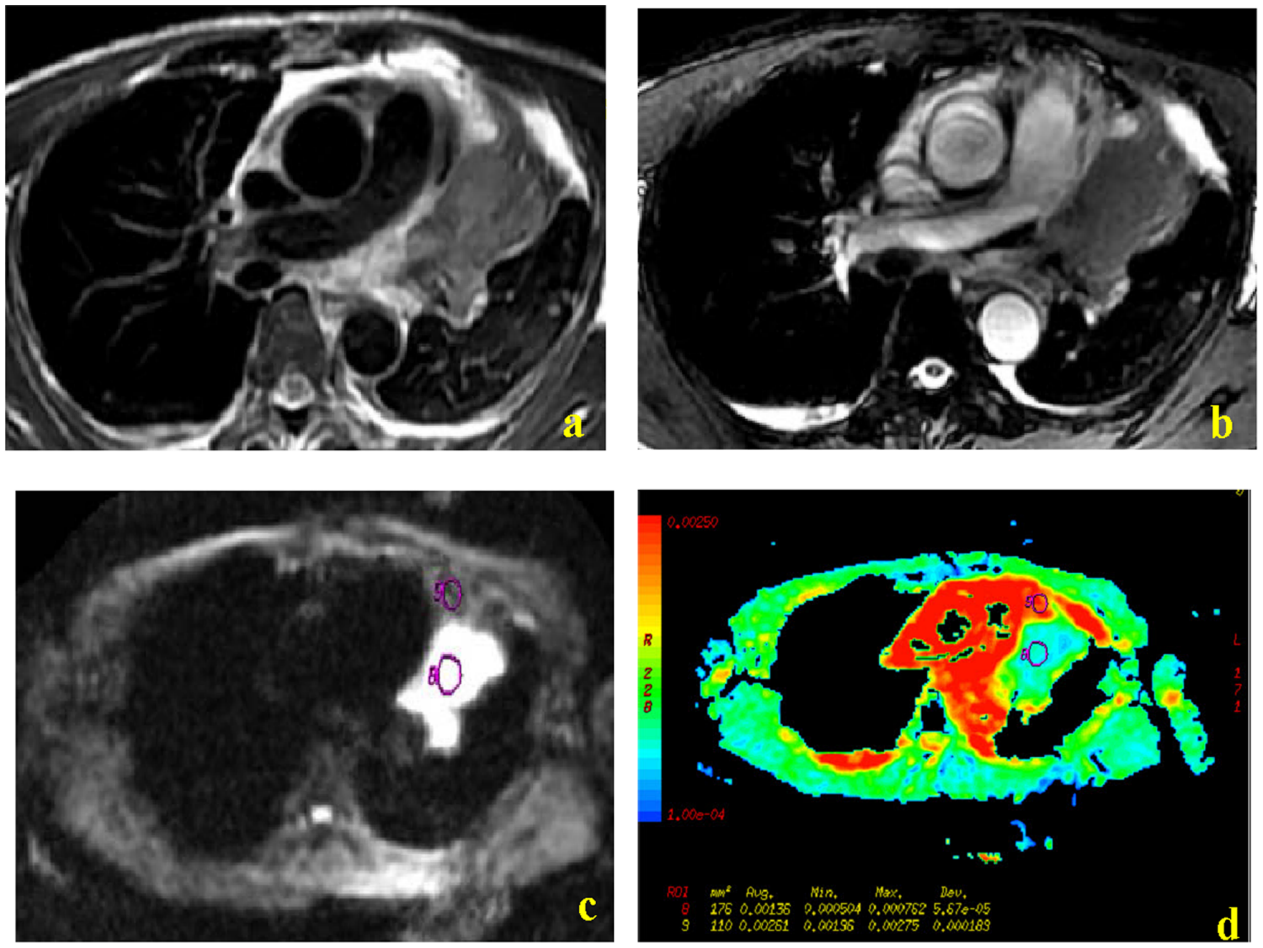
MR images of a 48-year-old man with squamous cell lung carcinoma. FRFSE T2W images and FIESTA images indicate clear hyperintensity of the tumor relative to the atelectasis (a and b, respectively). DW images obtained with a diffusion gradient of 500 s/mm^2^ allowed easy differentiation of tumor and atelectasis (c and d).

Figures 1, 2 and 3 show representative PET/CT and MR images. PET/CT imaging indicated that tumor masses had more FDG uptake than the atelectasis in all 38 patients, making them easily distinguishable (Fig. 1a, 2a). In contrast, the T1W MR images did not allow differentiation of tumor and atelectasis in any of the 38 patients (Fig. 1b, 2b). Conventional T2W images allowed differentiation of lung cancer and atelectasis in 76% of cases (29/38) (Fig. 1c, 1d, 2c, 2d, 3a, 3b). Among these 29 distinguishable cases, the signal intensity of the tumor was lower than the atelectasis in 28 cases (Fig. 1c, 1d, 2c, 2d), and higher than the atelectasis in one case (Fig. 3a, 3b). DW-MR images allowed easy differentiation of tumor and atelectasis in all 38 patients, and the tumor had higher signal intensity in all cases (Fig. 1e, 2e, 3c,). In addition, ADC maps clearly showed that the central lung carcinoma had lower mean ADC (indicated by hyperintensity) (Fig. 1f, 2f, 3d).

### CNR of Cancer and Atelectasis on Conventional T2W and DW-MR Images

**Figure 4:**
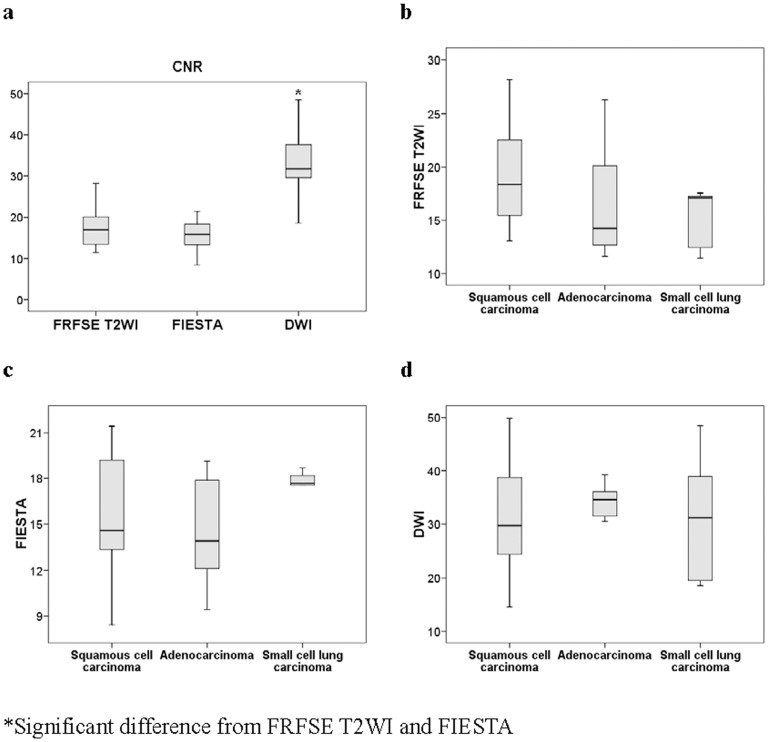
Comparison of contrast noise ratio (CNR) between cancer and atelectasis, and between different histological types of lung cancer. Differences of CNR between conventional FRFSE T2W, FIESTA, and DW-MR imaging of cancer and atelectasis (a). Differences of CNR between different histological types of cancer (b, c, and d).

The CNR of DW-MR images were clearly better than those from the conventional FRFSE T2WI and FIESTA MR images (*p*<0.0001 for both) (Figure 4a). However, the CNR values of the different histologic types of central lung carcinoma were not significantly different under these 3 methods (Figure 4b–d).

### ADC Mapping of Cancer and Atelectasis

**Figure 5:**
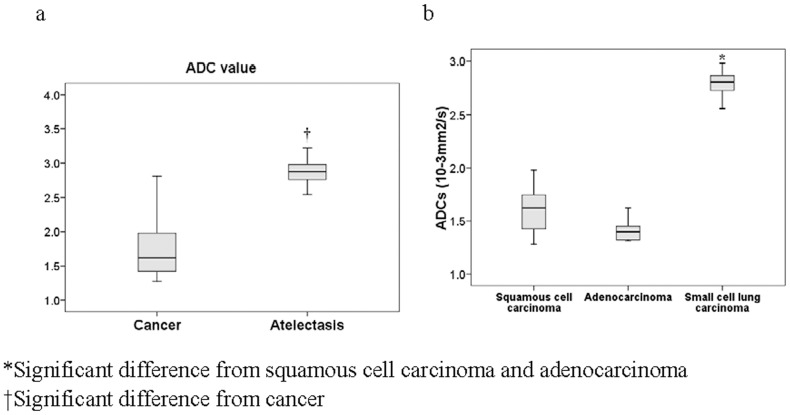
Comparison of apparent diffusion coefficients (10^−3 ^mm^2^/s) between cancer and atelectasis, and between different histological types of lung cancer. Differences of ADC between cancer and atelectasis (a). Differences of ADC between different histological types of central lung carcinoma (b). b-values were 0 and 500 s/mm^2^.

With a b-value of 500 s/mm^2^, the mean ADC for the central lung carcinoma was significantly less than that of the atelectasis (1.83±0.58 *vs.* 2.90±0.26 mm^2^/s, *p*<0.0001) (Figure 5a). Analysis of the different histologic types of central lung carcinomas indicated that the mean ADC of small cell lung carcinoma was significantly greater than those from squamous cell carcinoma and adenocarcinoma (*p*<0.0001 for both) (Figure 5b), but that squamous cell carcinoma and adenocarcinoma were not significantly different from each other.

## Discussion

We prospectively evaluated the performance of DW-MR imaging for the differentiation of central lung cancer and atelectasis in 38 consecutive lung cancer patients from three institutions and compared the performance of this method with conventional MR imaging and PET/CT imaging. PET/CT imaging and DW-MR both provided differentiation of tumor and atelectasis in all 38 patients, but conventional MR imaging did not perform as well. Thus, DW-MR imaging provides valuable information not obtained by conventional MR and has potential for clinical differentiation of central lung carcinoma from atelectasis.

Atelectasis is common in patients with central lung tumors. Lung cancer is routinely treated by surgery, radiation therapy, or chemotherapy, alone or in combination. Thus, differentiation of the tumor and atelectasis is important for assessment of the size and other characteristics of the tumor and for identification of tumor location, which is necessary for biopsy and targeted radiotherapy [2].

CT is the most widely available and commonly used non-invasive imaging method for patients with thoracic diseases. Previous studies indicated that dynamic enhanced CT scanning can differentiate tumor from atelectasis in about 80% of cases [5]. However, bolus-enhanced CT performs poorly in the differentiation of tumor and atelectasis [1]. PET/CT allows evaluation of the metabolism of endobronchial lesions [9], with increased FDG uptake at the site of obstruction, but this method is expensive and involves exposure to additional radiation.

Magnetic resonance imaging (MRI) is widely used and is a promising technique for tumor imaging because it does not employ ionizing radiation and it provides excellent soft tissue contrast and high spatial resolution [10], [11]. Tobler [5] retrospectively analyzed the ability of conventional MRI to distinguish atelectasis from tumors in the presence of a central bronchogenic carcinoma in 18 patients. Their results indicated that the T2W imaging sequences were much more informative than the T1W imaging sequences. This agrees with our results, in which T2W provided differentiation of tumor and atelectasis in 29 of 38 cases (76%), but T1W provided no such differentiation.

DW-MR imaging provides information on the molecular diffusion of water within tissues [2], [12]–[14]. This method can be performed quickly, there is no need for administration of exogenous contrast medium [15], [16], it yields information indicative of changes at the cellular level, and it provides unique insights about tumor cellularity and the integrity of cell membranes [15], [17]. Recent advances allow this technique to be used for tumor detection and characterization, distinguishing of tumor tissue from non-tumor tissue, and monitoring and predicting of treatment response.

DW-MR imaging of the lung must be included in the measurement of true whole-body DW-MR imaging [18], [19]. Nonetheless, following the improvement of the echo-planar technique, DW echo planar MR imaging with its speed and relatively high signal-to-noise ratio, can be successfully performed in thoracic lesions [15]. The signal intensity provided by DW-MR imaging can characterize tissue and provide information about cellular density. In addition, the ADC is associated with the cellular density of tumor, and reduced ADC occurs in most malignant tumors [13]. Our DW-MR images indicated that the central lung carcinoma had a hyper-intense signal relative to the atelectasis in all 38 cases. Our results also indicated that the CNR of DW-MR images were significantly higher than those from conventional T2W images. The increased contrast between cancer and atelectasis readily allowed visualization of the lung tumor.

The mean ADC is expected to be lower in viable tumor tissue, which is densely packed, than in tissues with less densely packed obstacles, such as tumor necrosis or benign tissue [20], [21]. Baysal et al [6] recently evaluated DW MR imaging for differentiation of atelectasis from central lung carcinoma for a b value of 1000 s/mm^2^. Their mean ADC for the central lung carcinomas with post-obstructive consolidations was 1.83±0.75×10^−3^ mm^2^/s, and the mean ADC for consolidations was 2.50±0.76×10^−3^ mm^2^/s (*p* = 0.003) [6]. In our study, the mean ADC values of the atelectasis were lower, but the mean ADC values of both the central lung carcinoma and atelectasis were still significantly different.

Matoba et al [21] prospectively evaluated DW MR imaging for characterization of lung carcinomas by use of the ADC, and reported that the mean ADC of adenocarcinomas was significantly higher than that of squamous cell carcinomas and large-cell carcinomas, and that the mean ADC value of well-differentiated adenocarcinomas was significantly higher than that of poorly differentiated adenocarcinomas. In contrast, our results indicated that small cell carcinoma had higher mean ADC than adenocarcinoma and squamous cell carcinoma. Thus, our results disagree with those of Matoba et al [22] but are consistent with those of with Baysal et al [6].

However, the ability of DW-MR imaging to be used for evaluation of thoracic lesions is hindered by image distortion because of its susceptibility to artifacts, including chemical shift artifacts, ghosts in the phase encoding direction, and respiratory and cardiac motions. Further studies are necessary to further assess the usefulness of DW-MR imaging and to determine whether DW-MR imaging can be used in clinical practice for the differentiation of central lung carcinoma and the accompanying atelectasis.

### Conclusion

In conclusion, the results of this study indicate that DW-MR imaging may be useful for the differentiation of central lung carcinoma and accompanying atelectasis. Although our sample size was relatively small, our results indicate that evaluation of DW-MR signal intensity distribution and ADC maps may aid in the choice of biopsy site and selection of the field to be used for radiotherapy.
